# Tris(*N*,*N*-di­methyl­anilinium) hexa­bromido­stannate(IV) bromide

**DOI:** 10.1107/S1600536813012403

**Published:** 2013-05-11

**Authors:** Hassen Chouaib, Slaheddine Kamoun, Hassine Ferid Ayedi

**Affiliations:** a Laboratoire de Génie des Matériaux et Environnement, École Nationale d’Ingénieurs de Sfax, BP 1173, Sfax, Tunisia

## Abstract

In the title compound, (C_8_H_12_N)_3_[SnBr_6_]Br, the anilinium N atom of one of the three unique cations exhibits flip-flop disorder with an 0.60:0.40 occupancy ratio. In the crystal, N—H⋯Br hydrogen bonds link the *N*,*N*-di­methyl­anilinium cations and both Br^−^ anions and [SnBr_6_]^2−^ dianions into a layered arrangement parallel to (001).

## Related literature

For related structures, see: Ali *et al.* (2008 ▶), Al-Far *et al.* (2009 ▶); Howie *et al.* (2009 ▶). For electric, magnetic and dielectric properties of related compounds, see: Hiraga *et al.* (2007 ▶); Karoui *et al.* (2013 ▶). 
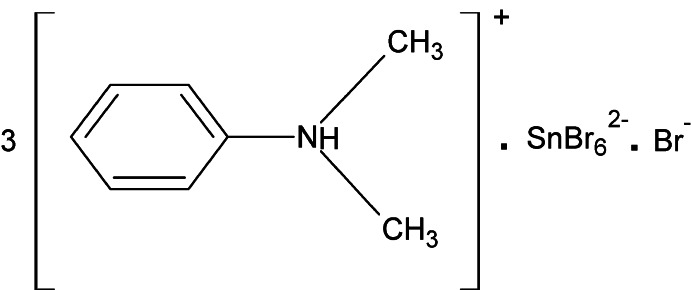


## Experimental

### 

#### Crystal data

(C_8_H_12_N)_3_[SnBr_6_]Br
*M* *_r_* = 1044.62
Monoclinic, 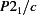

*a* = 14.3408 (6) Å
*b* = 9.1904 (4) Å
*c* = 26.4029 (12) Å
β = 93.451 (2)°
*V* = 3473.5 (3) Å^3^
*Z* = 4
Mo *K*α radiation
μ = 8.81 mm^−1^
*T* = 296 K
0.10 × 0.10 × 0.10 mm

#### Data collection

Bruker APEXII CCD diffractometer
Absorption correction: multi-scan (*SADABS*; Bruker, 2006 ▶) *T*_min_ = 0.415, *T*_max_ = 0.431
27006 measured reflections
6112 independent reflections
4555 reflections with *I* > 2σ(*I*)
*R*_int_ = 0.044

#### Refinement

*R*[*F* ^2^ > 2σ(*F* ^2^)] = 0.030
*wR*(*F* ^2^) = 0.064
*S* = 1.01
6112 reflections
333 parameters
7 restraints
H-atom parameters constrained
Δρ_max_ = 0.55 e Å^−3^
Δρ_min_ = −0.38 e Å^−3^

### 

Data collection: *APEX2* (Bruker, 2006 ▶); cell refinement: *SAINT* (Bruker, 2006 ▶); data reduction: *SAINT*; program(s) used to solve structure: *SHELXS97* (Sheldrick, 2008 ▶); program(s) used to refine structure: *SHELXL97* (Sheldrick, 2008 ▶); molecular graphics: *Mercury* (Macrae *et al.*, 2008 ▶); software used to prepare material for publication: *WinGX* (Farrugia, 2012 ▶) and *publCIF* (Westrip, 2010 ▶).

## Supplementary Material

Crystal structure: contains datablock(s) I, global. DOI: 10.1107/S1600536813012403/nk2205sup1.cif

Structure factors: contains datablock(s) I. DOI: 10.1107/S1600536813012403/nk2205Isup2.hkl

Supplementary material file. DOI: 10.1107/S1600536813012403/nk2205Isup3.cdx

Additional supplementary materials:  crystallographic information; 3D view; checkCIF report

## Figures and Tables

**Table 1:** Hydrogen-bond geometry (Å, °)

*D*—H⋯*A*	*D*—H	H⋯*A*	*D*⋯*A*	*D*—H⋯*A*
N11—H1⋯Br7^i^	0.91	2.38	3.269 (4)	165
N21—H2⋯Br7	0.91	2.30	3.196 (4)	168
N31*B*—H3*B*⋯Br5	0.91	2.74	3.631 (8)	167.1
N31*A*—H3*A*⋯Br4^ii^	0.83	2.56	3.352 (13)	159.7

Symmetry codes: (i) 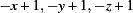; (ii) 
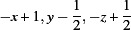
.

**Fig. 1.**
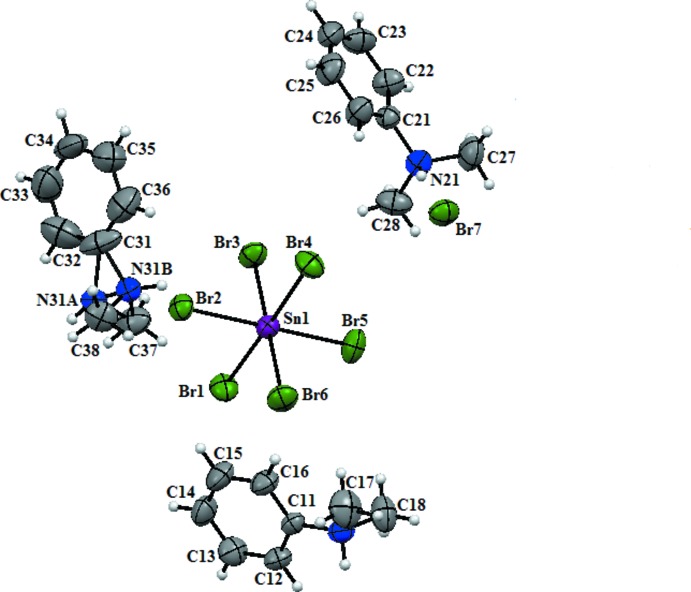
A view of the asymmetric unit of the title compound . Displacement ellipsoids are drawn at the 50% probability level.

**Fig. 2.**
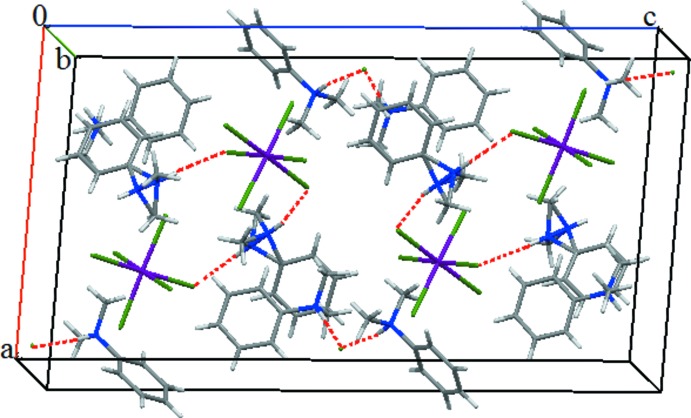
The crystal packing of the title compound showing the hydrogen bonding network as red dashed lines.

## supplementary crystallographic information

### Comment

Research in the field of organic-inorganic hybrid compounds is of great interest, because of their special magnetic (Hiraga *et al.*, 2007) and electronic (Karoui *et al.*, 2013) properties. The influence of the different organic cations is expected to affect the packing and then the specific properties. The title crystal structure contains three *N*,*N*-dimethylanilinium cations, one bromide ion and one isolated SnBr6 dianions, Fig.1. Each Sn site is surrounded by six Br ligands forming a distorted [SnBr_6_]^2-^ octahedron with Sn—Br bond lengths ranging from 2.5767 (6) Å to 2.6217 (6) Å. The relatively high values of C—N distances (> 1.5 Å) are due to the disorder of the nitrogen atom N(31). The π-π interactions between phenyl rings may be neglected (>4 Å); in fact the shortest distances between the centroids of the rings are: *Cg*2 ··· *Cg*1^i^ = 4.784 (4) Å; *Cg*1 ··· *Cg*3^ii^ = 4.993 (4) Å, *Cg*3 ··· *Cg*2^ii^ = 5.140 (3) Å (*Cg*1, *Cg*2 and *Cg*3 are the centroids of the C12–C16 and C21–C26 rings, C31—C36 rings, respectively; symmetry codes: (i) *x*,-1 + *y*,*z*; (ii)-*x*, 0.5 + *y*, 0.5 - *z*). The major contributions to the cohesion and the stability of the structure is the presence of N—H···Br hydrogen bonds which provide a linkage between *N*,*N*-dimethylanilinium cations and both Br- anions and [SnBr_6_]^2-^ dianions which include four relatively medium contacts, with H···Br and N..Br distances ranging from 2.30 Å to 2.74 Å and 3.196 (4) Å to 3.631 (8) Å, respectively (Fig. 2 and Table 1).

### Experimental

The title compound was prepared by refluxing during 5 h a solution of metallic tin (3 g, 25 mmol) in 40 ml an aqueous solution of hydrobromic acid, HBr 47%. To this solution, 9.5 ml (75 mmol) of a solution of *N*,*N*-dimethylaniline was added at reflux temperature. After a slow solvent evaporation yellow crystals suitable for X-ray analysis were obtained. They were washed with diethyl ether and dried over P_2_O_5_.

### Refinement

During refinement, the nitrogen atom N(31) showed a static flip-flop disorder. The disordered model was refined with fixed occupancy ratio 60:40 using the tools available in *SHELXL97* (Sheldrick, 2008): SADI for restraining and EADP to correlate anisotropic thermal parameters for related disordered atoms. All H atoms were geometrically positioned and treated as riding on their parent atoms, with C—H = 0.93 Å for the phenyl, 0.96 Å for the methyl and N—H= 0.91 Å with *U*_iso_(H)= 1.2 *U*_eq_(*C*-_phenyl_, N) or, 1.5Ueq(*C*-_methyl_).

### Crystal data

**Table:**

(C_8_H_12_N)_3_[SnBr_6_]Br	*F*(000) = 1984
*M**_r_* = 1044.62	Cell parameters from 57951 reflections
Monoclinic, *P*2_1_/*c*	*D*_x_ = 1.998 Mg m^−^^3^*D*_m_ = 2.009 Mg m^−^^3^*D*_m_ measured by Flotation
Hall symbol: -P 2ybc	Mo *K*α radiation, λ = 0.71073 Å
*a* = 14.3408 (6) Å	Cell parameters from 57951 reflections
*b* = 9.1904 (4) Å	θ = 1.6–25.0°
*c* = 26.4029 (12) Å	µ = 8.81 mm^−^^1^
β = 93.451 (2)°	*T* = 296 K
*V* = 3473.5 (3) Å^3^	Cube, yellow
*Z* = 4	0.10 × 0.10 × 0.10 mm

### Data collection

**Table:**

Bruker APEXII CCD diffractometer	6112 independent reflections
Radiation source: fine-focus sealed tube	4555 reflections with *I* > 2σ(*I*)
Graphite monochromator	*R*_int_ = 0.044
ω scans	θ_max_ = 25.0°, θ_min_ = 1.6°
Absorption correction: multi-scan (*SADABS*; Bruker, 2006)	*h* = −16→17
*T*_min_ = 0.415, *T*_max_ = 0.431	*k* = −10→10
27006 measured reflections	*l* = −31→31

### Refinement

**Table:**

Refinement on *F*^2^	Primary atom site location: structure-invariant direct methods
Least-squares matrix: full	Secondary atom site location: difference Fourier map
*R*[*F*^2^ > 2σ(*F*^2^)] = 0.030	Hydrogen site location: inferred from neighbouring sites
*wR*(*F*^2^) = 0.064	H-atom parameters constrained
*S* = 1.01	*w* = 1/[σ^2^(*F*_o_^2^) + (0.020*P*)^2^ + 4.9952*P*] where *P* = (*F*_o_^2^ + 2*F*_c_^2^)/3
6112 reflections	(Δ/σ)_max_ = 0.001
333 parameters	Δρ_max_ = 0.55 e Å^−^^3^
7 restraints	Δρ_min_ = −0.38 e Å^−^^3^

### Special details

**Table:**

Geometry. All e.s.d.'s (except the e.s.d. in the dihedral angle between two l.s. planes) are estimated using the full covariance matrix. The cell e.s.d.'s are taken into account individually in the estimation of e.s.d.'s in distances, angles and torsion angles; correlations between e.s.d.'s in cell parameters are only used when they are defined by crystal symmetry. An approximate (isotropic) treatment of cell e.s.d.'s is used for estimating e.s.d.'s involving l.s. planes.
Refinement. Refinement of *F*^2^ against ALL reflections. The weighted *R*-factor *wR* and goodness of fit *S* are based on *F*^2^, conventional *R*-factors *R* are based on *F*, with *F* set to zero for negative *F*^2^. The threshold expression of *F*^2^ > σ(*F*^2^) is used only for calculating *R*-factors(gt) *etc*. and is not relevant to the choice of reflections for refinement. *R*-factors based on *F*^2^ are statistically about twice as large as those based on *F*, and *R*- factors based on ALL data will be even larger.

### Fractional atomic coordinates and isotropic or equivalent isotropic displacement parameters (Å^2^)

**Table:**

	*x*	*y*	*z*	*U*_iso_*/*U*_eq_	Occ. (<1)
Sn1	0.34886 (2)	0.42038 (3)	0.357239 (11)	0.03803 (9)	
Br1	0.51100 (3)	0.36489 (6)	0.32373 (2)	0.05700 (15)	
Br2	0.27708 (3)	0.21753 (5)	0.297717 (19)	0.05008 (13)	
Br3	0.18550 (3)	0.47638 (6)	0.38796 (2)	0.06065 (15)	
Br4	0.42257 (4)	0.62582 (6)	0.415392 (19)	0.06096 (16)	
Br5	0.32156 (4)	0.61078 (6)	0.283898 (19)	0.05471 (14)	
Br6	0.37920 (4)	0.23426 (6)	0.42947 (2)	0.06195 (15)	
Br7	0.05993 (4)	0.72433 (6)	0.48704 (2)	0.06009 (15)	
C11	0.8147 (3)	0.4670 (5)	0.40030 (16)	0.0408 (11)	
C12	0.9067 (3)	0.4520 (5)	0.38938 (18)	0.0490 (12)	
H12	0.9543	0.4607	0.4147	0.059*	
C13	0.9264 (4)	0.4237 (6)	0.3399 (2)	0.0600 (14)	
H13	0.9882	0.4142	0.3315	0.072*	
C14	0.8561 (4)	0.4095 (6)	0.30307 (19)	0.0651 (15)	
H14	0.8703	0.3888	0.2699	0.078*	
C15	0.7657 (4)	0.4254 (7)	0.3145 (2)	0.0671 (16)	
H15	0.7181	0.4172	0.2891	0.080*	
C16	0.7444 (3)	0.4537 (6)	0.36346 (19)	0.0575 (14)	
H16	0.6825	0.4637	0.3715	0.069*	
C17	0.7015 (4)	0.4466 (7)	0.4685 (2)	0.086 (2)	
H17A	0.6945	0.4708	0.5035	0.128*	
H17B	0.6977	0.3430	0.4643	0.128*	
H17C	0.6528	0.4924	0.4477	0.128*	
C18	0.8038 (4)	0.6560 (6)	0.4652 (2)	0.0769 (18)	
H18A	0.7906	0.6722	0.5000	0.115*	
H18B	0.7606	0.7107	0.4435	0.115*	
H18C	0.8664	0.6868	0.4599	0.115*	
N11	0.7940 (3)	0.4989 (5)	0.45325 (14)	0.0525 (10)	
H1	0.8380	0.4517	0.4734	0.063*	
C21	0.0426 (3)	0.9402 (5)	0.35440 (17)	0.0438 (11)	
C22	0.0362 (4)	1.0562 (6)	0.3223 (2)	0.0590 (14)	
H22	0.0750	1.1366	0.3273	0.071*	
C23	−0.0299 (4)	1.0508 (7)	0.2821 (2)	0.0689 (16)	
H23	−0.0354	1.1288	0.2597	0.083*	
C24	−0.0864 (4)	0.9350 (7)	0.2747 (2)	0.0653 (15)	
H24	−0.1309	0.9341	0.2475	0.078*	
C25	−0.0788 (4)	0.8201 (7)	0.3066 (2)	0.0735 (17)	
H25	−0.1179	0.7401	0.3013	0.088*	
C26	−0.0128 (4)	0.8212 (6)	0.3471 (2)	0.0634 (15)	
H26	−0.0064	0.7418	0.3689	0.076*	
C27	0.1164 (5)	1.0776 (6)	0.4274 (2)	0.0774 (18)	
H27A	0.1623	1.0696	0.4552	0.116*	
H27B	0.0563	1.0964	0.4402	0.116*	
H27C	0.1328	1.1562	0.4057	0.116*	
C28	0.2073 (4)	0.8948 (6)	0.3829 (2)	0.0707 (16)	
H28A	0.2500	0.8944	0.4123	0.106*	
H28B	0.2285	0.9626	0.3584	0.106*	
H28C	0.2041	0.7992	0.3683	0.106*	
N21	0.1128 (3)	0.9387 (4)	0.39786 (15)	0.0509 (10)	
H2	0.0942	0.8687	0.4194	0.061*	
C32	0.4009 (5)	0.4525 (8)	0.0832 (3)	0.107 (3)	
H32	0.4589	0.4171	0.0751	0.128*	
C38	0.4210 (4)	0.2539 (6)	0.1905 (2)	0.0648 (15)	
C37	0.5277 (4)	0.4612 (7)	0.1808 (3)	0.0710 (17)	
C31	0.3753 (4)	0.4520 (7)	0.1324 (3)	0.084 (2)	
N31A	0.4589 (7)	0.3584 (11)	0.1545 (4)	0.044 (2)	0.40
C34	0.2539 (5)	0.5579 (7)	0.0589 (3)	0.100 (3)	
H34	0.2123	0.5950	0.0338	0.120*	
C35	0.2300 (5)	0.5551 (8)	0.1080 (3)	0.100 (2)	
H35	0.1719	0.5895	0.1163	0.119*	
C36	0.2906 (5)	0.5022 (8)	0.1448 (3)	0.097 (2)	
H36	0.2744	0.5003	0.1783	0.117*	
C33	0.3390 (6)	0.5064 (9)	0.0464 (3)	0.109 (3)	
H33	0.3548	0.5080	0.0128	0.131*	
N31B	0.4261 (5)	0.4111 (8)	0.1831 (3)	0.0515 (18)	0.60
H3B	0.4100	0.4645	0.2103	0.13 (5)*	0.60
H3A	0.5006	0.3114	0.1410	0.18 (11)*	0.40
H37A	0.5243	0.5588	0.1673	0.14 (3)*	
H37B	0.5753	0.4084	0.1646	0.21 (5)*	
H37C	0.5437	0.4663	0.2167	0.15 (3)*	
H38A	0.4597	0.1850	0.1747	0.31 (7)*	
H38B	0.3564	0.2317	0.1825	0.10 (2)*	
H38C	0.4338	0.2541	0.2263	0.29 (6)*	

### Atomic displacement parameters (Å^2^)

**Table:**

	*U*^11^	*U*^22^	*U*^33^	*U*^12^	*U*^13^	*U*^23^
Sn1	0.03490 (16)	0.04135 (18)	0.03801 (17)	−0.00518 (14)	0.00366 (13)	0.00125 (14)
Br1	0.0349 (3)	0.0660 (3)	0.0708 (4)	−0.0059 (2)	0.0090 (2)	−0.0079 (3)
Br2	0.0450 (3)	0.0501 (3)	0.0548 (3)	−0.0096 (2)	−0.0002 (2)	−0.0069 (2)
Br3	0.0456 (3)	0.0628 (3)	0.0754 (4)	0.0049 (3)	0.0187 (3)	−0.0003 (3)
Br4	0.0764 (4)	0.0629 (3)	0.0441 (3)	−0.0256 (3)	0.0081 (3)	−0.0089 (3)
Br5	0.0653 (3)	0.0499 (3)	0.0481 (3)	−0.0041 (3)	−0.0039 (2)	0.0104 (2)
Br6	0.0702 (3)	0.0619 (3)	0.0533 (3)	0.0010 (3)	−0.0001 (3)	0.0163 (3)
Br7	0.0630 (3)	0.0667 (4)	0.0504 (3)	0.0031 (3)	0.0019 (2)	0.0128 (3)
C11	0.042 (3)	0.044 (3)	0.036 (2)	−0.002 (2)	−0.002 (2)	0.005 (2)
C12	0.041 (3)	0.057 (3)	0.049 (3)	−0.002 (2)	−0.005 (2)	0.006 (3)
C13	0.051 (3)	0.072 (4)	0.058 (3)	−0.003 (3)	0.014 (3)	0.005 (3)
C14	0.077 (4)	0.079 (4)	0.039 (3)	−0.001 (3)	0.002 (3)	−0.005 (3)
C15	0.061 (4)	0.086 (4)	0.052 (3)	−0.011 (3)	−0.013 (3)	−0.006 (3)
C16	0.045 (3)	0.071 (4)	0.054 (3)	−0.003 (3)	−0.007 (2)	0.000 (3)
C17	0.083 (4)	0.103 (5)	0.075 (4)	−0.012 (4)	0.037 (3)	−0.001 (4)
C18	0.079 (4)	0.072 (4)	0.082 (4)	−0.014 (3)	0.016 (3)	−0.027 (3)
N11	0.048 (2)	0.063 (3)	0.047 (2)	0.009 (2)	0.0001 (19)	0.003 (2)
C21	0.039 (2)	0.049 (3)	0.043 (3)	−0.001 (2)	0.002 (2)	−0.002 (2)
C22	0.059 (3)	0.053 (3)	0.065 (3)	−0.009 (3)	0.003 (3)	0.015 (3)
C23	0.072 (4)	0.070 (4)	0.065 (4)	0.010 (3)	−0.002 (3)	0.022 (3)
C24	0.059 (3)	0.080 (4)	0.055 (3)	0.010 (3)	−0.009 (3)	−0.010 (3)
C25	0.077 (4)	0.066 (4)	0.075 (4)	−0.017 (3)	−0.011 (3)	−0.011 (4)
C26	0.082 (4)	0.045 (3)	0.062 (4)	−0.012 (3)	−0.010 (3)	0.005 (3)
C27	0.100 (5)	0.062 (4)	0.069 (4)	−0.012 (3)	−0.009 (3)	−0.016 (3)
C28	0.049 (3)	0.071 (4)	0.092 (4)	0.003 (3)	0.005 (3)	0.017 (3)
N21	0.057 (3)	0.047 (2)	0.049 (2)	−0.006 (2)	0.001 (2)	0.004 (2)
C32	0.093 (5)	0.083 (5)	0.144 (8)	0.024 (4)	0.010 (6)	0.015 (5)
C38	0.070 (4)	0.055 (4)	0.071 (4)	−0.008 (3)	0.015 (3)	0.018 (3)
C37	0.061 (4)	0.063 (4)	0.087 (5)	−0.015 (3)	−0.014 (3)	0.007 (3)
C31	0.067 (4)	0.080 (5)	0.101 (5)	−0.026 (4)	−0.038 (4)	0.038 (4)
N31A	0.040 (6)	0.045 (6)	0.048 (6)	0.004 (5)	−0.002 (5)	0.006 (5)
C34	0.123 (6)	0.068 (5)	0.101 (6)	0.021 (4)	−0.067 (5)	−0.007 (4)
C35	0.091 (5)	0.098 (6)	0.106 (6)	0.021 (4)	−0.016 (5)	−0.012 (5)
C36	0.101 (6)	0.109 (6)	0.079 (5)	−0.019 (5)	−0.017 (4)	−0.009 (4)
C33	0.161 (8)	0.093 (6)	0.072 (5)	0.023 (6)	−0.005 (5)	−0.005 (4)
N31B	0.049 (4)	0.055 (5)	0.050 (4)	0.005 (4)	−0.001 (4)	−0.005 (4)

### Geometric parameters (Å, º)

**Table:**

Sn1—Br3	2.5767 (6)	C25—H25	0.9300
Sn1—Br6	2.5792 (6)	C26—H26	0.9300
Sn1—Br1	2.5879 (6)	C27—N21	1.495 (6)
Sn1—Br2	2.6096 (6)	C27—H27A	0.9600
Sn1—Br4	2.6161 (6)	C27—H27B	0.9600
Sn1—Br5	2.6217 (6)	C27—H27C	0.9600
C11—C16	1.363 (6)	C28—N21	1.490 (6)
C11—C12	1.375 (6)	C28—H28A	0.9600
C11—N11	1.476 (5)	C28—H28B	0.9600
C12—C13	1.378 (7)	C28—H28C	0.9600
C12—H12	0.9300	N21—H2	0.9100
C13—C14	1.364 (7)	C32—C33	1.370 (7)
C13—H13	0.9300	C32—C31	1.371 (6)
C14—C15	1.357 (7)	C32—H32	0.9300
C14—H14	0.9300	C38—N31B	1.460 (9)
C15—C16	1.371 (7)	C38—N31A	1.478 (11)
C15—H15	0.9300	C38—H38A	0.954
C16—H16	0.9300	C38—H38B	0.960
C17—N11	1.488 (7)	C38—H38C	0.951
C17—H17A	0.9600	C37—N31A	1.507 (11)
C17—H17B	0.9600	C37—N31B	1.532 (9)
C17—H17C	0.9600	C37—H37A	0.966
C18—N11	1.483 (7)	C37—H37B	0.960
C18—H18A	0.9600	C37—H37C	0.962
C18—H18B	0.9600	C31—C36	1.356 (7)
C18—H18C	0.9600	C31—N31B	1.531 (9)
N11—H1	0.9100	C31—N31A	1.560 (12)
C21—C26	1.358 (7)	N31A—N31B	1.034 (12)
C21—C22	1.362 (7)	N31A—H3A	0.834
C21—N21	1.480 (6)	C34—C35	1.361 (7)
C22—C23	1.380 (7)	C34—C33	1.367 (7)
C22—H22	0.9300	C34—H34	0.9300
C23—C24	1.345 (8)	C35—C36	1.354 (7)
C23—H23	0.9300	C35—H35	0.9300
C24—C25	1.351 (8)	C36—H36	0.9300
C24—H24	0.9300	C33—H33	0.9300
C25—C26	1.386 (7)	N31B—H3B	0.912
			
Br3—Sn1—Br6	90.91 (2)	H27B—C27—H27C	109.5
Br3—Sn1—Br1	178.36 (2)	N21—C28—H28A	109.5
Br6—Sn1—Br1	90.40 (2)	N21—C28—H28B	109.5
Br3—Sn1—Br2	89.948 (19)	H28A—C28—H28B	109.5
Br6—Sn1—Br2	90.82 (2)	N21—C28—H28C	109.5
Br1—Sn1—Br2	89.042 (19)	H28A—C28—H28C	109.5
Br3—Sn1—Br4	90.80 (2)	H28B—C28—H28C	109.5
Br6—Sn1—Br4	90.03 (2)	C21—N21—C28	112.6 (4)
Br1—Sn1—Br4	90.19 (2)	C21—N21—C27	113.4 (4)
Br2—Sn1—Br4	178.86 (2)	C28—N21—C27	111.5 (4)
Br3—Sn1—Br5	90.06 (2)	C21—N21—H2	106.3
Br6—Sn1—Br5	178.87 (2)	C28—N21—H2	106.3
Br1—Sn1—Br5	88.64 (2)	C27—N21—H2	106.3
Br2—Sn1—Br5	89.773 (19)	C33—C32—C31	118.3 (6)
Br4—Sn1—Br5	89.37 (2)	C33—C32—H32	120.9
C16—C11—C12	121.3 (4)	C31—C32—H32	120.9
C16—C11—N11	120.8 (4)	N31B—C38—N31A	41.2 (5)
C12—C11—N11	118.0 (4)	N31B—C38—H38A	124.3
C11—C12—C13	118.2 (4)	N31A—C38—H38A	84.5
C11—C12—H12	120.9	N31B—C38—H38B	103.6
C13—C12—H12	120.9	N31A—C38—H38B	112.5
C14—C13—C12	120.6 (5)	H38A—C38—H38B	109.9
C14—C13—H13	119.7	N31B—C38—H38C	97.1
C12—C13—H13	119.7	N31A—C38—H38C	125.6
C15—C14—C13	120.5 (5)	H38A—C38—H38C	110.7
C15—C14—H14	119.8	H38B—C38—H38C	110.1
C13—C14—H14	119.8	N31A—C37—N31B	39.8 (5)
C14—C15—C16	119.9 (5)	N31A—C37—H37A	113.3
C14—C15—H15	120.0	N31B—C37—H37A	105.5
C16—C15—H15	120.0	N31A—C37—H37B	86.6
C11—C16—C15	119.6 (5)	N31B—C37—H37B	124.7
C11—C16—H16	120.2	H37A—C37—H37B	109.0
C15—C16—H16	120.2	N31A—C37—H37C	126.6
N11—C17—H17A	109.5	N31B—C37—H37C	98.6
N11—C17—H17B	109.5	H37A—C37—H37C	108.9
H17A—C17—H17B	109.5	H37B—C37—H37C	109.3
N11—C17—H17C	109.5	C36—C31—C32	121.5 (6)
H17A—C17—H17C	109.5	C36—C31—N31B	105.0 (7)
H17B—C17—H17C	109.5	C32—C31—N31B	133.4 (7)
N11—C18—H18A	109.5	C36—C31—N31A	140.9 (8)
N11—C18—H18B	109.5	C32—C31—N31A	96.4 (7)
H18A—C18—H18B	109.5	N31B—C31—N31A	39.1 (4)
N11—C18—H18C	109.5	N31B—N31A—C38	68.5 (8)
H18A—C18—H18C	109.5	N31B—N31A—C37	71.5 (8)
H18B—C18—H18C	109.5	C38—N31A—C37	111.4 (8)
C11—N11—C18	111.9 (4)	N31B—N31A—C31	68.9 (8)
C11—N11—C17	115.2 (4)	C38—N31A—C31	107.2 (7)
C18—N11—C17	109.3 (4)	C37—N31A—C31	107.1 (7)
C11—N11—H1	106.6	N31B—N31A—H3A	157.3 (12)
C18—N11—H1	106.6	C38—N31A—H3A	103.8 (9)
C17—N11—H1	106.6	C37—N31A—H3A	93.1 (8)
C26—C21—C22	121.7 (5)	C31—N31A—H3A	133.0 (11)
C26—C21—N21	117.8 (4)	C35—C34—C33	120.2 (6)
C22—C21—N21	120.4 (4)	C35—C34—H34	119.9
C21—C22—C23	117.9 (5)	C33—C34—H34	119.9
C21—C22—H22	121.1	C36—C35—C34	120.1 (7)
C23—C22—H22	121.1	C36—C35—H35	119.9
C24—C23—C22	121.4 (5)	C34—C35—H35	119.9
C24—C23—H23	119.3	C35—C36—C31	119.7 (7)
C22—C23—H23	119.3	C35—C36—H36	120.2
C23—C24—C25	120.1 (5)	C31—C36—H36	120.2
C23—C24—H24	119.9	C34—C33—C32	120.2 (7)
C25—C24—H24	119.9	C34—C33—H33	119.9
C24—C25—C26	120.1 (5)	C32—C33—H33	119.9
C24—C25—H25	119.9	N31A—N31B—C38	70.3 (8)
C26—C25—H25	119.9	N31A—N31B—C31	72.0 (8)
C21—C26—C25	118.8 (5)	C38—N31B—C31	109.6 (6)
C21—C26—H26	120.6	N31A—N31B—C37	68.8 (7)
C25—C26—H26	120.6	C38—N31B—C37	110.9 (6)
N21—C27—H27A	109.5	C31—N31B—C37	107.3 (6)
N21—C27—H27B	109.5	N31A—N31B—H3B	167.5
H27A—C27—H27B	109.5	C38—N31B—H3B	114.1
N21—C27—H27C	109.5	C31—N31B—H3B	115.3
H27A—C27—H27C	109.5	C37—N31B—H3B	98.9
			
C16—C11—C12—C13	0.3 (7)	C32—C31—N31A—N31B	163.7 (8)
N11—C11—C12—C13	−179.4 (4)	C36—C31—N31A—C38	27.9 (14)
C11—C12—C13—C14	−0.7 (8)	C32—C31—N31A—C38	−138.4 (8)
C12—C13—C14—C15	1.1 (9)	N31B—C31—N31A—C38	58.0 (8)
C13—C14—C15—C16	−1.0 (9)	C36—C31—N31A—C37	−91.7 (11)
C12—C11—C16—C15	−0.3 (8)	C32—C31—N31A—C37	102.0 (8)
N11—C11—C16—C15	179.4 (5)	N31B—C31—N31A—C37	−61.7 (8)
C14—C15—C16—C11	0.6 (9)	C33—C34—C35—C36	0.5 (12)
C16—C11—N11—C18	−97.6 (5)	C34—C35—C36—C31	−0.1 (12)
C12—C11—N11—C18	82.1 (5)	C32—C31—C36—C35	−0.5 (11)
C16—C11—N11—C17	28.1 (7)	N31B—C31—C36—C35	176.5 (7)
C12—C11—N11—C17	−152.2 (5)	N31A—C31—C36—C35	−164.4 (9)
C26—C21—C22—C23	1.0 (8)	C35—C34—C33—C32	−0.4 (12)
N21—C21—C22—C23	179.2 (4)	C31—C32—C33—C34	−0.1 (12)
C21—C22—C23—C24	0.1 (8)	C37—N31A—N31B—C38	−123.1 (5)
C22—C23—C24—C25	−0.7 (9)	C31—N31A—N31B—C38	119.5 (5)
C23—C24—C25—C26	0.1 (9)	C38—N31A—N31B—C31	−119.5 (5)
C22—C21—C26—C25	−1.6 (8)	C37—N31A—N31B—C31	117.5 (5)
N21—C21—C26—C25	−179.8 (5)	C38—N31A—N31B—C37	123.1 (5)
C24—C25—C26—C21	1.0 (9)	C31—N31A—N31B—C37	−117.5 (5)
C26—C21—N21—C28	96.9 (5)	N31A—C38—N31B—C31	61.5 (8)
C22—C21—N21—C28	−81.4 (6)	N31A—C38—N31B—C37	−56.7 (8)
C26—C21—N21—C27	−135.4 (5)	C36—C31—N31B—N31A	160.9 (8)
C22—C21—N21—C27	46.3 (6)	C32—C31—N31B—N31A	−22.6 (11)
C33—C32—C31—C36	0.5 (11)	C36—C31—N31B—C38	100.4 (7)
C33—C32—C31—N31B	−175.4 (7)	C32—C31—N31B—C38	−83.1 (10)
C33—C32—C31—N31A	170.4 (7)	N31A—C31—N31B—C38	−60.5 (8)
N31B—C38—N31A—C37	58.6 (8)	C36—C31—N31B—C37	−139.0 (6)
N31B—C38—N31A—C31	−58.3 (8)	C32—C31—N31B—C37	37.4 (11)
N31B—C37—N31A—C38	−56.9 (8)	N31A—C31—N31B—C37	60.0 (8)
N31B—C37—N31A—C31	60.0 (8)	N31A—C37—N31B—C38	57.6 (8)
C36—C31—N31A—N31B	−30.0 (13)	N31A—C37—N31B—C31	−62.1 (8)

### Hydrogen-bond geometry (Å, º)

**Table:**

*D*—H···*A*	*D*—H	H···*A*	*D*···*A*	*D*—H···*A*
N11—H1···Br7^i^	0.91	2.38	3.269 (4)	165
N21—H2···Br7	0.91	2.30	3.196 (4)	168
N31*B*—H3*B*···Br5	0.91	2.74	3.631 (8)	167.1
N31*A*—H3*A*···Br4^ii^	0.83	2.56	3.352 (13)	159.7

Symmetry codes: (i) −*x*+1, −*y*+1, −*z*+1; (ii) −*x*+1, *y*−1/2, −*z*+1/2.
